# (*R*,*R*)-*N*,*N*′-Bis(ferrocenylmeth­yl)cyclo­hexane-1,2-diamine

**DOI:** 10.1107/S1600536809025057

**Published:** 2009-07-04

**Authors:** YingChun Wang

**Affiliations:** a Ordered Matter Science Research Center, College of Chemistry and Chemical Engineering, Southeast University, Nanjing 210096, People’s Republic of China

## Abstract

In the structure of the title compound, [Fe_2_(C_5_H_5_)_2_(C_18_H_24_N_2_)], the cyclo­hexane ring has a chair configuration and the two ferrocenemethyl­amino groups are bonded to it equatorially, as expected. The configuration of the two ferrocence nuclei may be due to intra­molecular N—H⋯N hydrogen bonding involving the two NH groups.

## Related literature

For the applications of ferrocene derivatives, see: Yang *et al.* (2002 ▶); Roberto *et al.* (2000 ▶); Beer (1998 ▶). For the crystal structures of related compounds, see: Hess *et al.* (1999 ▶); Base *et al.* (2002 ▶). For the synthetic strategy, see: Cho *et al.* (1999 ▶); Sutcliffe (2002 ▶).
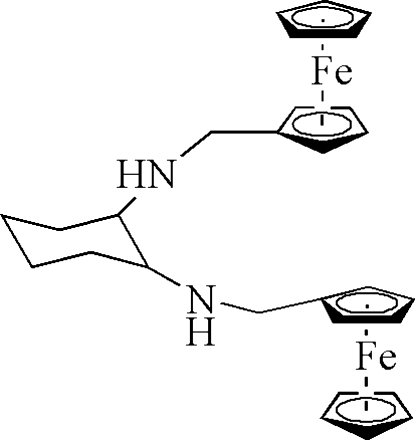


## Experimental

### 

#### Crystal data

[Fe_2_(C_5_H_5_)_2_(C_18_H_24_N_2_)]
*M* *_r_* = 510.27
Monoclinic, 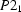

*a* = 5.9384 (7) Å
*b* = 10.5310 (11) Å
*c* = 19.173 (3) Å
β = 90.989 (10)°
*V* = 1198.9 (3) Å^3^
*Z* = 2
Mo *K*α radiation
μ = 1.23 mm^−1^
*T* = 293 K
0.15 × 0.12 × 0.10 mm

#### Data collection

Rigaku SCXmini diffractometer
Absorption correction: multi-scan (*CrystalClear*; Rigaku, 2005 ▶) *T*_min_ = 0.856, *T*_max_ = 1.000 (expected range = 0.757–0.884)
12081 measured reflections
5474 independent reflections
4717 reflections with *I* > 2σ(*I*)
*R*_int_ = 0.044

#### Refinement

*R*[*F* ^2^ > 2σ(*F* ^2^)] = 0.042
*wR*(*F* ^2^) = 0.087
*S* = 1.06
5474 reflections
290 parameters
1 restraint
H-atom parameters constrained
Δρ_max_ = 0.27 e Å^−3^
Δρ_min_ = −0.45 e Å^−3^
Absolute structure: Flack (1983 ▶), 2571 Friedel pairs
Flack parameter: 0.035 (19)

### 

Data collection: *CrystalClear* (Rigaku, 2005 ▶); cell refinement: *CrystalClear*; data reduction: *CrystalClear*; program(s) used to solve structure: *SHELXS97* (Sheldrick, 2008 ▶); program(s) used to refine structure: *SHELXL97* (Sheldrick, 2008 ▶); molecular graphics: *SHELXTL* (Sheldrick, 2008 ▶); software used to prepare material for publication: *SHELXTL*.

## Supplementary Material

Crystal structure: contains datablocks I, global. DOI: 10.1107/S1600536809025057/su2124sup1.cif

Structure factors: contains datablocks I. DOI: 10.1107/S1600536809025057/su2124Isup2.hkl

Additional supplementary materials:  crystallographic information; 3D view; checkCIF report

## Figures and Tables

**Table 1:** Hydrogen-bond geometry (Å, °)

*D*—H⋯*A*	*D*—H	H⋯*A*	*D*⋯*A*	*D*—H⋯*A*
N1—H1*A*⋯N2	0.90	2.36	2.848 (4)	114

**Fig. 1.**
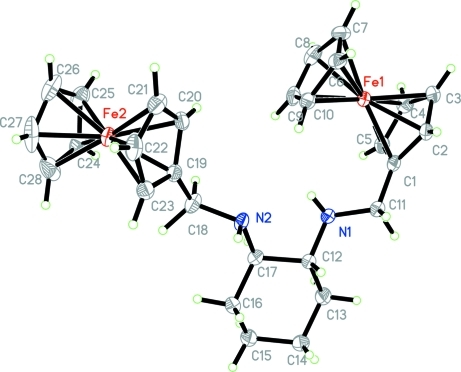
A view of the molecular structure of the title compound, with the displacement ellipsoids were drawn at the 30% probability level.

**Fig. 2.**
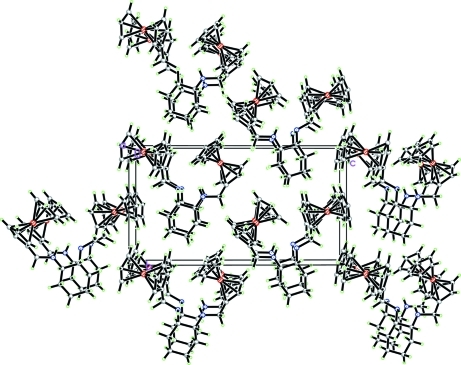
A crystal packing diagram of the title compound, viewed along the a axis.

## supplementary crystallographic information

### Comment

The chemistry of ferrocene has received much attention as both ferrocene and its derivatives have applications in many fields. For example, ferrocene has been employed as a marker for the electrochemical detection of amino acids in selective anion sensors (Beer *et al.*, 1998). It also has applications in catalysis (Yang *et al.*, 2002), non-linear optical (NLO) materials (Roberto *et al.*, 2000) and medicinal materials. As part of our continuing studies on new ferrocene compounds, we report herein on the crystal structure of the title compound,

*R*,*R*)—*N*^1^,*N*^2^-Cyclohexane-1,2-\ bis((ferrocenylmethylene)amine, (I).

The molecular structure of the title compound is illustrated in Fig. 1. It consists of (1*R*,2*R*)-cyclohexane-1,2-diamine units, in which the hydrogen atoms of the two amine groups have been substituted by ferrocenylmethyl units. The cyclohexane ring has a chair conformation and the two ferrocenemethylamino groups are equatorially bonded to it, with torsion angles C1—C11—N1—C12 and C19—C18—N2—C17 being 178.4 (3) and 168.3 (3)°, respectively. The bond lengths and angles and the conformation of the ferrocenyl group, are similar to those observed in other ferrocenemethylamino derivatives (Hess *et al.*, 1999; Base *et al.*, 2002). The Cp ring planes (C6—C10 and C19—C23), are inclined to one another by 48.84 (15)°. The intramolecular Fe—Fe distance is 7.6054 (11) Å, while the nearest intermolecular Fe—Fe separation is 5.9384 (7) Å. The orientation of the two ferrocence nuclei is probably due to the presence of the intramolecular N1—H1···N22 hydrogen bond (Table 1).

The crystal packing of the title compound is illustrated in Fig. 2.

### Experimental

The title compound was synthesized by reducing the corresponding Schiff base (*R*,*R*)—N1,N2-Cyclohexane-1,2-bis((ferrocenylmethylene)) (5 mmol) (Cho *et al.*,  1999), with sodium borohydride (40 mmol) in methanol, in an ice bath. 40 ml NaOH solution (1 mol.*L*^-1^) was added and the mixture was stirred at rt for an 1 h (Sutcliffe *et al.*, 2002)). The mixture was extracted with CHCl_3_ and the solvent removed under vacumn. The crude product was purified by recrystalization from acetone. Red crystals, suitable for X-ray diffraction analysis, were obtained by slow evaporation of a methanol solution after 3 days.

### Refinement

The H-atoms were included in calculated positions and treated as riding atoms: N—H = 0.86 Å, C—H = 0.93 - 0.97 Å, with U_iso_(H) = 1.2U_eq_(parent N- or C-atom).

### Crystal data

**Table:**

[Fe_2_(C_5_H_5_)_2_(C_18_H_24_N_2_)]	*F*(000) = 536
*M**_r_* = 510.27	*D*_x_ = 1.413 Mg m^−^^3^
Monoclinic, *P*2_1_	Mo *K*α radiation, λ = 0.71073 Å
Hall symbol: P 2yb	Cell parameters from 3140 reflections
*a* = 5.9384 (7) Å	θ = 3.2–27.5°
*b* = 10.5310 (11) Å	µ = 1.23 mm^−^^1^
*c* = 19.173 (3) Å	*T* = 293 K
β = 90.989 (10)°	Block, yellow
*V* = 1198.9 (3) Å^3^	0.15 × 0.12 × 0.10 mm
*Z* = 2	

### Data collection

**Table:**

Rigaku SCXmini diffractometer	5474 independent reflections
Radiation source: fine-focus sealed tube	4717 reflections with *I* > 2σ(*I*)
graphite	*R*_int_ = 0.044
Detector resolution: 13.6612 pixels mm^-1^	θ_max_ = 27.5°, θ_min_ = 3.2°
ω scans	*h* = −7→7
Absorption correction: multi-scan (*CrystalClear*; Rigaku, 2005)	*k* = −13→13
*T*_min_ = 0.856, *T*_max_ = 1.000	*l* = −24→24
12081 measured reflections	

### Refinement

**Table:**

Refinement on *F*^2^	Hydrogen site location: inferred from neighbouring sites
Least-squares matrix: full	H-atom parameters constrained
*R*[*F*^2^ > 2σ(*F*^2^)] = 0.042	*w* = 1/[σ^2^(*F*_o_^2^) + (0.0266*P*)^2^] where *P* = (*F*_o_^2^ + 2*F*_c_^2^)/3
*wR*(*F*^2^) = 0.087	(Δ/σ)_max_ = 0.005
*S* = 1.06	Δρ_max_ = 0.27 e Å^−^^3^
5474 reflections	Δρ_min_ = −0.45 e Å^−^^3^
290 parameters	Extinction correction: *SHELXL97* (Sheldrick, 2008), Fc^*^=kFc[1+0.001xFc^2^λ^3^/sin(2θ)]^-1/4^
1 restraint	Extinction coefficient: 0.0039 (6)
Primary atom site location: structure-invariant direct methods	Absolute structure: Flack (1983), 2567 Friedel pairs
Secondary atom site location: difference Fourier map	Flack parameter: 0.035 (19)

### Special details

**Table:**

Geometry. Bond distances, angles etc. have been calculated using the rounded fractional coordinates. All su's are estimated from the variances of the (full) variance-covariance matrix. The cell esds are taken into account in the estimation of distances, angles and torsion angles
Refinement. Refinement of *F*^2^ against ALL reflections. The weighted *R*-factor *wR* and goodness of fit *S* are based on *F*^2^, conventional *R*-factors *R* are based on *F*, with *F* set to zero for negative *F*^2^. The threshold expression of *F*^2^ > σ(*F*^2^) is used only for calculating *R*-factors(gt) *etc*. and is not relevant to the choice of reflections for refinement. *R*-factors based on *F*^2^ are statistically about twice as large as those based on *F*, and *R*- factors based on ALL data will be even larger.

### Fractional atomic coordinates and isotropic or equivalent isotropic displacement parameters (Å^2^)

**Table:**

	*x*	*y*	*z*	*U*_iso_*/*U*_eq_	
Fe1	−0.30550 (8)	0.14014 (4)	0.41771 (2)	0.0319 (2)	
Fe2	0.31701 (8)	0.05780 (4)	0.07731 (2)	0.0359 (2)	
N1	−0.1496 (5)	0.4290 (3)	0.33760 (15)	0.0420 (10)	
N2	0.1430 (5)	0.3628 (3)	0.22723 (15)	0.0424 (10)	
C1	−0.2745 (6)	0.3278 (3)	0.44519 (18)	0.0346 (10)	
C2	−0.2197 (6)	0.2484 (3)	0.50262 (18)	0.0379 (11)	
C3	−0.4105 (7)	0.1713 (4)	0.5177 (2)	0.0439 (12)	
C4	−0.5809 (6)	0.2026 (4)	0.4685 (2)	0.0455 (11)	
C5	−0.4994 (6)	0.2994 (3)	0.42391 (19)	0.0392 (11)	
C6	−0.0664 (6)	0.0033 (3)	0.40032 (19)	0.0433 (11)	
C7	−0.2839 (6)	−0.0511 (3)	0.4033 (2)	0.0482 (11)	
C8	−0.4153 (7)	0.0049 (4)	0.3496 (2)	0.0527 (14)	
C9	−0.2840 (7)	0.0948 (4)	0.3144 (2)	0.0484 (14)	
C10	−0.0653 (6)	0.0945 (3)	0.34622 (18)	0.0403 (11)	
C11	−0.1262 (6)	0.4267 (3)	0.41367 (18)	0.0421 (11)	
C12	−0.0144 (7)	0.5256 (3)	0.30284 (19)	0.0412 (11)	
C13	−0.1359 (8)	0.6533 (4)	0.3064 (2)	0.0589 (14)	
C14	−0.0159 (9)	0.7556 (4)	0.2652 (2)	0.069 (2)	
C15	0.0105 (10)	0.7148 (4)	0.1895 (2)	0.0685 (16)	
C16	0.1400 (8)	0.5900 (4)	0.1867 (2)	0.0578 (14)	
C17	0.0245 (7)	0.4849 (4)	0.2277 (2)	0.0427 (11)	
C18	0.1208 (6)	0.2940 (4)	0.16031 (19)	0.0484 (12)	
C19	0.2803 (6)	0.1851 (3)	0.15736 (18)	0.0363 (11)	
C20	0.2432 (6)	0.0588 (4)	0.18120 (17)	0.0405 (11)	
C21	0.4394 (7)	−0.0130 (4)	0.1695 (2)	0.0498 (12)	
C22	0.5983 (6)	0.0673 (4)	0.13930 (18)	0.0508 (13)	
C23	0.5017 (6)	0.1894 (4)	0.13173 (19)	0.0418 (11)	
C24	0.0702 (7)	0.0889 (4)	0.0051 (2)	0.0582 (14)	
C25	0.0701 (7)	−0.0383 (4)	0.0239 (2)	0.0531 (16)	
C26	0.2822 (8)	−0.0894 (5)	0.0087 (2)	0.0665 (17)	
C27	0.4131 (7)	0.0072 (7)	−0.0203 (2)	0.080 (2)	
C28	0.2810 (9)	0.1171 (6)	−0.0231 (2)	0.077 (2)	
H1A	−0.10720	0.35250	0.32140	0.0500*	
H2A	−0.07450	0.24620	0.52770	0.0450*	
H2C	0.29010	0.37760	0.23580	0.0510*	
H3A	−0.42060	0.10780	0.55490	0.0530*	
H4A	−0.73120	0.16430	0.46570	0.0550*	
H5A	−0.58320	0.33930	0.38530	0.0470*	
H6A	0.06100	−0.01700	0.43150	0.0520*	
H7A	−0.33290	−0.11700	0.43580	0.0580*	
H8A	−0.57340	−0.01500	0.33880	0.0640*	
H9A	−0.33370	0.14780	0.27500	0.0580*	
H10A	0.06260	0.14750	0.33290	0.0480*	
H11A	−0.16520	0.50940	0.43220	0.0500*	
H11B	0.02950	0.40940	0.42660	0.0500*	
H12A	0.13150	0.53290	0.32720	0.0490*	
H13A	−0.28810	0.64350	0.28800	0.0710*	
H13B	−0.14530	0.67960	0.35470	0.0710*	
H14A	0.13140	0.77130	0.28610	0.0830*	
H14B	−0.10150	0.83390	0.26700	0.0830*	
H15A	−0.13680	0.70420	0.16760	0.0820*	
H15B	0.09100	0.77980	0.16410	0.0820*	
H16A	0.29080	0.60290	0.20570	0.0690*	
H16B	0.15340	0.56370	0.13840	0.0690*	
H17A	−0.12390	0.47120	0.20580	0.0510*	
H18A	0.15040	0.35190	0.12220	0.0580*	
H18B	−0.03220	0.26300	0.15460	0.0580*	
H20A	0.10410	0.02690	0.20170	0.0480*	
H21A	0.46100	−0.10290	0.18080	0.0600*	
H22A	0.75080	0.04300	0.12580	0.0610*	
H23A	0.57550	0.26400	0.11190	0.0500*	
H24A	−0.05610	0.14810	0.00960	0.0700*	
H25A	−0.05550	−0.08430	0.04460	0.0640*	
H26A	0.33020	−0.17720	0.01690	0.0800*	
H27A	0.56820	−0.00110	−0.03640	0.0960*	
H28A	0.32780	0.19970	−0.04130	0.0920*	

### Atomic displacement parameters (Å^2^)

**Table:**

	*U*^11^	*U*^22^	*U*^33^	*U*^12^	*U*^13^	*U*^23^
Fe1	0.0361 (3)	0.0313 (3)	0.0283 (3)	0.0006 (2)	0.0029 (2)	−0.0026 (2)
Fe2	0.0349 (3)	0.0429 (3)	0.0298 (3)	0.0027 (3)	−0.0002 (2)	−0.0080 (2)
N1	0.063 (2)	0.0298 (15)	0.0337 (17)	−0.0066 (14)	0.0129 (14)	−0.0055 (13)
N2	0.0559 (19)	0.0376 (16)	0.0339 (16)	0.0043 (14)	0.0064 (14)	−0.0109 (13)
C1	0.0415 (18)	0.0341 (18)	0.0285 (18)	0.0034 (15)	0.0058 (14)	−0.0078 (14)
C2	0.046 (2)	0.041 (2)	0.0267 (18)	−0.0007 (17)	0.0006 (15)	−0.0047 (14)
C3	0.057 (2)	0.043 (2)	0.032 (2)	−0.0020 (19)	0.0139 (17)	−0.0036 (16)
C4	0.0378 (18)	0.049 (2)	0.050 (2)	−0.0018 (17)	0.0097 (17)	−0.0082 (18)
C5	0.0350 (17)	0.0418 (19)	0.041 (2)	0.0060 (15)	0.0048 (15)	−0.0042 (16)
C6	0.049 (2)	0.0328 (17)	0.048 (2)	0.0058 (16)	0.0003 (17)	−0.0036 (16)
C7	0.061 (2)	0.0311 (19)	0.053 (2)	−0.0061 (17)	0.013 (2)	0.0020 (17)
C8	0.046 (2)	0.054 (2)	0.058 (3)	−0.004 (2)	−0.002 (2)	−0.025 (2)
C9	0.066 (3)	0.050 (2)	0.0290 (19)	0.016 (2)	−0.0021 (17)	−0.0073 (17)
C10	0.0485 (19)	0.0336 (17)	0.0393 (19)	0.0017 (16)	0.0127 (16)	−0.0028 (15)
C11	0.053 (2)	0.0363 (18)	0.037 (2)	−0.0050 (17)	0.0055 (16)	−0.0060 (15)
C12	0.060 (2)	0.0322 (19)	0.0317 (19)	−0.0089 (17)	0.0062 (17)	−0.0033 (15)
C13	0.096 (3)	0.039 (2)	0.042 (2)	0.005 (2)	0.014 (2)	−0.001 (2)
C14	0.130 (5)	0.030 (2)	0.047 (3)	−0.006 (3)	0.015 (3)	−0.0015 (19)
C15	0.130 (4)	0.035 (2)	0.041 (2)	−0.003 (3)	0.019 (3)	0.003 (2)
C16	0.089 (3)	0.043 (2)	0.042 (2)	−0.010 (2)	0.021 (2)	0.0010 (18)
C17	0.057 (2)	0.0331 (19)	0.038 (2)	−0.0017 (18)	0.0055 (17)	−0.0046 (16)
C18	0.057 (2)	0.047 (2)	0.041 (2)	0.0072 (19)	−0.0060 (19)	−0.0137 (18)
C19	0.0371 (18)	0.0406 (19)	0.0314 (19)	−0.0026 (16)	0.0031 (15)	−0.0064 (15)
C20	0.0461 (18)	0.046 (2)	0.0297 (17)	−0.0076 (19)	0.0055 (14)	−0.0043 (17)
C21	0.061 (2)	0.044 (2)	0.044 (2)	0.013 (2)	−0.0119 (19)	−0.0073 (18)
C22	0.0384 (18)	0.075 (3)	0.039 (2)	0.011 (2)	−0.0018 (15)	−0.011 (2)
C23	0.0385 (18)	0.050 (2)	0.037 (2)	−0.0132 (17)	0.0007 (15)	−0.0054 (16)
C24	0.061 (2)	0.064 (3)	0.049 (2)	0.014 (2)	−0.019 (2)	−0.012 (2)
C25	0.049 (2)	0.057 (3)	0.053 (3)	−0.006 (2)	−0.0048 (18)	−0.015 (2)
C26	0.069 (3)	0.069 (3)	0.061 (3)	0.020 (3)	−0.012 (3)	−0.038 (3)
C27	0.042 (2)	0.157 (6)	0.041 (3)	−0.008 (3)	0.008 (2)	−0.037 (3)
C28	0.102 (4)	0.097 (4)	0.030 (2)	−0.041 (4)	−0.013 (2)	0.010 (2)

### Geometric parameters (Å, °)

**Table:**

Fe1—C1	2.053 (3)	C18—C19	1.489 (5)
Fe1—C2	2.045 (3)	C19—C20	1.425 (5)
Fe1—C3	2.053 (4)	C19—C23	1.412 (5)
Fe1—C4	2.028 (4)	C20—C21	1.410 (6)
Fe1—C5	2.039 (3)	C21—C22	1.400 (6)
Fe1—C6	2.054 (3)	C22—C23	1.414 (6)
Fe1—C7	2.037 (3)	C24—C28	1.404 (7)
Fe1—C8	2.032 (4)	C24—C25	1.387 (6)
Fe1—C9	2.044 (4)	C25—C26	1.405 (6)
Fe1—C10	2.052 (4)	C26—C27	1.401 (8)
Fe2—C19	2.052 (3)	C27—C28	1.399 (9)
Fe2—C20	2.047 (3)	C2—H2A	0.9800
Fe2—C21	2.041 (4)	C3—H3A	0.9800
Fe2—C22	2.036 (4)	C4—H4A	0.9800
Fe2—C23	2.043 (4)	C5—H5A	0.9800
Fe2—C24	2.025 (4)	C6—H6A	0.9800
Fe2—C25	2.042 (4)	C7—H7A	0.9800
Fe2—C26	2.041 (5)	C8—H8A	0.9800
Fe2—C27	2.037 (4)	C9—H9A	0.9800
Fe2—C28	2.032 (4)	C10—H10A	0.9800
N1—C11	1.463 (4)	C11—H11A	0.9700
N1—C12	1.464 (5)	C11—H11B	0.9700
N2—C17	1.466 (5)	C12—H12A	0.9800
N2—C18	1.477 (5)	C13—H13A	0.9700
N1—H1A	0.9000	C13—H13B	0.9700
N2—H2C	0.9000	C14—H14A	0.9700
C1—C5	1.422 (5)	C14—H14B	0.9700
C1—C11	1.498 (5)	C15—H15A	0.9700
C1—C2	1.416 (5)	C15—H15B	0.9700
C2—C3	1.428 (5)	C16—H16A	0.9700
C3—C4	1.411 (5)	C16—H16B	0.9700
C4—C5	1.421 (5)	C17—H17A	0.9800
C6—C10	1.414 (5)	C18—H18A	0.9700
C6—C7	1.415 (5)	C18—H18B	0.9700
C7—C8	1.410 (5)	C20—H20A	0.9800
C8—C9	1.406 (6)	C21—H21A	0.9800
C9—C10	1.425 (5)	C22—H22A	0.9800
C12—C17	1.525 (5)	C23—H23A	0.9800
C12—C13	1.528 (6)	C24—H24A	0.9800
C13—C14	1.521 (6)	C25—H25A	0.9800
C14—C15	1.524 (6)	C26—H26A	0.9800
C15—C16	1.524 (6)	C27—H27A	0.9800
C16—C17	1.527 (6)	C28—H28A	0.9800
			
C1—Fe1—C2	40.43 (13)	N1—C12—C17	109.2 (3)
C1—Fe1—C3	68.51 (15)	C12—C13—C14	112.0 (4)
C1—Fe1—C4	68.52 (15)	C13—C14—C15	110.6 (4)
C1—Fe1—C5	40.66 (14)	C14—C15—C16	109.7 (3)
C1—Fe1—C6	131.14 (14)	C15—C16—C17	112.1 (4)
C1—Fe1—C7	168.82 (15)	N2—C17—C12	109.5 (3)
C1—Fe1—C8	150.00 (15)	N2—C17—C16	114.4 (3)
C1—Fe1—C9	117.83 (15)	C12—C17—C16	111.1 (3)
C1—Fe1—C10	109.67 (13)	N2—C18—C19	111.3 (3)
C2—Fe1—C3	40.78 (15)	Fe2—C19—C18	127.4 (3)
C2—Fe1—C4	68.20 (15)	Fe2—C19—C20	69.46 (19)
C2—Fe1—C5	68.17 (14)	C20—C19—C23	107.0 (3)
C2—Fe1—C6	110.91 (14)	C18—C19—C23	125.8 (3)
C2—Fe1—C7	130.07 (14)	Fe2—C19—C23	69.5 (2)
C2—Fe1—C8	167.21 (15)	C18—C19—C20	127.2 (3)
C2—Fe1—C9	152.01 (15)	Fe2—C20—C21	69.6 (2)
C2—Fe1—C10	119.72 (14)	C19—C20—C21	108.5 (3)
C3—Fe1—C4	40.44 (16)	Fe2—C20—C19	69.86 (19)
C3—Fe1—C5	68.48 (16)	Fe2—C21—C20	70.1 (2)
C3—Fe1—C6	119.06 (16)	Fe2—C21—C22	69.7 (2)
C3—Fe1—C7	107.79 (16)	C20—C21—C22	107.9 (4)
C3—Fe1—C8	127.81 (16)	Fe2—C22—C21	70.1 (2)
C3—Fe1—C9	165.40 (17)	Fe2—C22—C23	70.0 (2)
C3—Fe1—C10	152.51 (15)	C21—C22—C23	108.5 (3)
C4—Fe1—C5	40.90 (15)	Fe2—C23—C22	69.4 (2)
C4—Fe1—C6	150.58 (15)	C19—C23—C22	108.3 (3)
C4—Fe1—C7	116.05 (16)	Fe2—C23—C19	70.2 (2)
C4—Fe1—C8	106.47 (16)	C25—C24—C28	108.0 (4)
C4—Fe1—C9	127.28 (16)	Fe2—C24—C25	70.7 (2)
C4—Fe1—C10	166.52 (15)	Fe2—C24—C28	70.0 (2)
C5—Fe1—C6	168.25 (14)	Fe2—C25—C26	69.8 (3)
C5—Fe1—C7	149.10 (14)	C24—C25—C26	108.2 (4)
C5—Fe1—C8	116.02 (16)	Fe2—C25—C24	69.4 (2)
C5—Fe1—C9	107.07 (16)	Fe2—C26—C25	69.9 (3)
C5—Fe1—C10	129.14 (14)	Fe2—C26—C27	69.7 (3)
C6—Fe1—C7	40.46 (14)	C25—C26—C27	108.0 (4)
C6—Fe1—C8	67.61 (15)	Fe2—C27—C26	70.1 (2)
C6—Fe1—C9	67.91 (16)	Fe2—C27—C28	69.7 (3)
C6—Fe1—C10	40.27 (14)	C26—C27—C28	107.5 (4)
C7—Fe1—C8	40.56 (16)	Fe2—C28—C27	70.1 (3)
C7—Fe1—C9	68.44 (16)	C24—C28—C27	108.3 (5)
C7—Fe1—C10	68.39 (14)	Fe2—C28—C24	69.5 (2)
C8—Fe1—C9	40.37 (16)	Fe1—C2—H2A	126.00
C8—Fe1—C10	68.02 (15)	C1—C2—H2A	126.00
C9—Fe1—C10	40.73 (15)	C3—C2—H2A	126.00
C19—Fe2—C20	40.68 (15)	Fe1—C3—H3A	126.00
C19—Fe2—C21	68.39 (15)	C2—C3—H3A	126.00
C19—Fe2—C22	68.16 (15)	C4—C3—H3A	126.00
C19—Fe2—C23	40.35 (14)	Fe1—C4—H4A	126.00
C19—Fe2—C24	108.70 (15)	C3—C4—H4A	126.00
C19—Fe2—C25	127.88 (15)	C5—C4—H4A	126.00
C19—Fe2—C26	165.27 (16)	Fe1—C5—H5A	126.00
C19—Fe2—C27	153.3 (2)	C1—C5—H5A	126.00
C19—Fe2—C28	119.8 (2)	C4—C5—H5A	126.00
C20—Fe2—C21	40.36 (16)	Fe1—C6—H6A	126.00
C20—Fe2—C22	67.60 (14)	C7—C6—H6A	126.00
C20—Fe2—C23	67.76 (15)	C10—C6—H6A	126.00
C20—Fe2—C24	119.99 (15)	Fe1—C7—H7A	126.00
C20—Fe2—C25	109.06 (15)	C6—C7—H7A	126.00
C20—Fe2—C26	127.59 (17)	C8—C7—H7A	126.00
C20—Fe2—C27	164.5 (2)	Fe1—C8—H8A	126.00
C20—Fe2—C28	154.1 (2)	C7—C8—H8A	125.00
C21—Fe2—C22	40.17 (16)	C9—C8—H8A	125.00
C21—Fe2—C23	68.00 (16)	Fe1—C9—H9A	126.00
C21—Fe2—C24	153.37 (17)	C8—C9—H9A	126.00
C21—Fe2—C25	119.68 (16)	C10—C9—H9A	126.00
C21—Fe2—C26	108.14 (17)	Fe1—C10—H10A	126.00
C21—Fe2—C27	126.8 (2)	C6—C10—H10A	126.00
C21—Fe2—C28	164.40 (19)	C9—C10—H10A	126.00
C22—Fe2—C23	40.58 (16)	N1—C11—H11A	109.00
C22—Fe2—C24	165.45 (17)	N1—C11—H11B	109.00
C22—Fe2—C25	153.11 (17)	C1—C11—H11A	109.00
C22—Fe2—C26	119.11 (18)	C1—C11—H11B	109.00
C22—Fe2—C27	108.01 (15)	H11A—C11—H11B	108.00
C22—Fe2—C28	127.59 (19)	N1—C12—H12A	109.00
C23—Fe2—C24	127.97 (16)	C13—C12—H12A	109.00
C23—Fe2—C25	165.20 (16)	C17—C12—H12A	109.00
C23—Fe2—C26	153.11 (17)	C12—C13—H13A	109.00
C23—Fe2—C27	119.36 (19)	C12—C13—H13B	109.00
C23—Fe2—C28	108.8 (2)	C14—C13—H13A	109.00
C24—Fe2—C25	39.88 (17)	C14—C13—H13B	109.00
C24—Fe2—C26	67.56 (18)	H13A—C13—H13B	108.00
C24—Fe2—C27	67.98 (17)	C13—C14—H14A	109.00
C24—Fe2—C28	40.49 (19)	C13—C14—H14B	109.00
C25—Fe2—C26	40.25 (18)	C15—C14—H14A	110.00
C25—Fe2—C27	67.65 (18)	C15—C14—H14B	110.00
C25—Fe2—C28	67.3 (2)	H14A—C14—H14B	108.00
C26—Fe2—C27	40.2 (2)	C14—C15—H15A	110.00
C26—Fe2—C28	67.4 (2)	C14—C15—H15B	110.00
C27—Fe2—C28	40.2 (2)	C16—C15—H15A	110.00
C11—N1—C12	115.0 (3)	C16—C15—H15B	110.00
C17—N2—C18	113.6 (3)	H15A—C15—H15B	108.00
C11—N1—H1A	108.00	C15—C16—H16A	109.00
C12—N1—H1A	108.00	C15—C16—H16B	109.00
C17—N2—H2C	108.00	C17—C16—H16A	109.00
C18—N2—H2C	108.00	C17—C16—H16B	109.00
C2—C1—C11	126.6 (3)	H16A—C16—H16B	108.00
C5—C1—C11	125.9 (3)	N2—C17—H17A	107.00
Fe1—C1—C5	69.15 (18)	C12—C17—H17A	107.00
Fe1—C1—C11	128.1 (2)	C16—C17—H17A	107.00
Fe1—C1—C2	69.48 (18)	N2—C18—H18A	109.00
C2—C1—C5	107.5 (3)	N2—C18—H18B	109.00
Fe1—C2—C1	70.09 (19)	C19—C18—H18A	109.00
Fe1—C2—C3	69.9 (2)	C19—C18—H18B	109.00
C1—C2—C3	108.7 (3)	H18A—C18—H18B	108.00
Fe1—C3—C2	69.3 (2)	Fe2—C20—H20A	126.00
Fe1—C3—C4	68.8 (2)	C19—C20—H20A	126.00
C2—C3—C4	107.1 (3)	C21—C20—H20A	126.00
Fe1—C4—C5	70.0 (2)	Fe2—C21—H21A	126.00
C3—C4—C5	108.8 (3)	C20—C21—H21A	126.00
Fe1—C4—C3	70.7 (2)	C22—C21—H21A	126.00
C1—C5—C4	107.8 (3)	Fe2—C22—H22A	126.00
Fe1—C5—C4	69.1 (2)	C21—C22—H22A	126.00
Fe1—C5—C1	70.19 (19)	C23—C22—H22A	126.00
Fe1—C6—C7	69.12 (19)	Fe2—C23—H23A	126.00
C7—C6—C10	108.7 (3)	C19—C23—H23A	126.00
Fe1—C6—C10	69.79 (19)	C22—C23—H23A	126.00
Fe1—C7—C8	69.5 (2)	Fe2—C24—H24A	126.00
C6—C7—C8	107.2 (3)	C25—C24—H24A	126.00
Fe1—C7—C6	70.42 (19)	C28—C24—H24A	126.00
Fe1—C8—C7	69.9 (2)	Fe2—C25—H25A	126.00
Fe1—C8—C9	70.3 (2)	C24—C25—H25A	126.00
C7—C8—C9	109.1 (4)	C26—C25—H25A	126.00
Fe1—C9—C8	69.4 (2)	Fe2—C26—H26A	126.00
C8—C9—C10	107.6 (3)	C25—C26—H26A	126.00
Fe1—C9—C10	70.0 (2)	C27—C26—H26A	126.00
Fe1—C10—C6	69.9 (2)	Fe2—C27—H27A	126.00
Fe1—C10—C9	69.3 (2)	C26—C27—H27A	126.00
C6—C10—C9	107.5 (3)	C28—C27—H27A	126.00
N1—C11—C1	111.6 (3)	Fe2—C28—H28A	126.00
C13—C12—C17	111.7 (3)	C24—C28—H28A	126.00
N1—C12—C13	109.2 (3)	C27—C28—H28A	126.00
			
C2—Fe1—C1—C5	−119.1 (3)	C26—Fe2—C21—C20	−127.3 (3)
C2—Fe1—C1—C11	121.0 (4)	C26—Fe2—C21—C22	113.9 (3)
C3—Fe1—C1—C2	37.6 (2)	C27—Fe2—C21—C20	−167.9 (3)
C3—Fe1—C1—C5	−81.6 (2)	C27—Fe2—C21—C22	73.3 (3)
C3—Fe1—C1—C11	158.5 (4)	C19—Fe2—C22—C21	81.9 (2)
C4—Fe1—C1—C2	81.2 (2)	C19—Fe2—C22—C23	−37.4 (2)
C4—Fe1—C1—C5	−37.9 (2)	C20—Fe2—C22—C21	37.9 (2)
C4—Fe1—C1—C11	−157.9 (3)	C20—Fe2—C22—C23	−81.5 (2)
C5—Fe1—C1—C2	119.1 (3)	C21—Fe2—C22—C23	−119.4 (3)
C5—Fe1—C1—C11	−119.9 (4)	C23—Fe2—C22—C21	119.4 (3)
C6—Fe1—C1—C2	−72.9 (3)	C25—Fe2—C22—C21	−50.3 (4)
C6—Fe1—C1—C5	168.0 (2)	C25—Fe2—C22—C23	−169.7 (3)
C6—Fe1—C1—C11	48.1 (4)	C26—Fe2—C22—C21	−83.8 (3)
C8—Fe1—C1—C2	167.0 (3)	C26—Fe2—C22—C23	156.8 (2)
C8—Fe1—C1—C5	47.9 (4)	C27—Fe2—C22—C21	−126.3 (3)
C8—Fe1—C1—C11	−72.0 (5)	C27—Fe2—C22—C23	114.4 (3)
C9—Fe1—C1—C2	−156.9 (2)	C28—Fe2—C22—C21	−166.5 (3)
C9—Fe1—C1—C5	84.0 (2)	C28—Fe2—C22—C23	74.1 (3)
C9—Fe1—C1—C11	−36.0 (4)	C19—Fe2—C23—C22	119.4 (3)
C10—Fe1—C1—C2	−113.1 (2)	C20—Fe2—C23—C19	−38.4 (2)
C10—Fe1—C1—C5	127.8 (2)	C20—Fe2—C23—C22	81.1 (2)
C10—Fe1—C1—C11	7.9 (3)	C21—Fe2—C23—C19	−82.1 (2)
C1—Fe1—C2—C3	119.7 (3)	C21—Fe2—C23—C22	37.3 (2)
C3—Fe1—C2—C1	−119.7 (3)	C22—Fe2—C23—C19	−119.4 (3)
C4—Fe1—C2—C1	−82.0 (2)	C24—Fe2—C23—C19	73.1 (3)
C4—Fe1—C2—C3	37.7 (2)	C24—Fe2—C23—C22	−167.5 (2)
C5—Fe1—C2—C1	−37.8 (2)	C26—Fe2—C23—C19	−169.0 (3)
C5—Fe1—C2—C3	81.9 (2)	C26—Fe2—C23—C22	−49.6 (4)
C6—Fe1—C2—C1	129.6 (2)	C27—Fe2—C23—C19	157.0 (3)
C6—Fe1—C2—C3	−110.7 (2)	C27—Fe2—C23—C22	−83.6 (3)
C7—Fe1—C2—C1	171.6 (2)	C28—Fe2—C23—C19	114.2 (2)
C7—Fe1—C2—C3	−68.7 (3)	C28—Fe2—C23—C22	−126.4 (2)
C9—Fe1—C2—C1	47.6 (4)	C19—Fe2—C24—C25	−127.3 (2)
C9—Fe1—C2—C3	167.3 (3)	C19—Fe2—C24—C28	114.3 (3)
C10—Fe1—C2—C1	85.9 (2)	C20—Fe2—C24—C25	−84.1 (3)
C10—Fe1—C2—C3	−154.4 (2)	C20—Fe2—C24—C28	157.5 (3)
C1—Fe1—C3—C2	−37.3 (2)	C21—Fe2—C24—C25	−48.4 (5)
C1—Fe1—C3—C4	81.7 (3)	C21—Fe2—C24—C28	−166.8 (4)
C2—Fe1—C3—C4	118.9 (3)	C23—Fe2—C24—C25	−168.1 (2)
C4—Fe1—C3—C2	−118.9 (3)	C23—Fe2—C24—C28	73.5 (3)
C5—Fe1—C3—C2	−81.1 (2)	C25—Fe2—C24—C28	−118.4 (4)
C5—Fe1—C3—C4	37.8 (2)	C26—Fe2—C24—C25	37.5 (2)
C6—Fe1—C3—C2	88.9 (2)	C26—Fe2—C24—C28	−80.9 (3)
C6—Fe1—C3—C4	−152.2 (2)	C27—Fe2—C24—C25	81.0 (3)
C7—Fe1—C3—C2	131.5 (2)	C27—Fe2—C24—C28	−37.4 (4)
C7—Fe1—C3—C4	−109.5 (2)	C28—Fe2—C24—C25	118.4 (4)
C8—Fe1—C3—C2	172.0 (2)	C19—Fe2—C25—C24	72.7 (3)
C8—Fe1—C3—C4	−69.1 (3)	C19—Fe2—C25—C26	−167.8 (2)
C10—Fe1—C3—C2	54.3 (4)	C20—Fe2—C25—C24	114.3 (2)
C10—Fe1—C3—C4	173.2 (3)	C20—Fe2—C25—C26	−126.2 (3)
C1—Fe1—C4—C3	−81.7 (2)	C21—Fe2—C25—C24	157.3 (2)
C1—Fe1—C4—C5	37.7 (2)	C21—Fe2—C25—C26	−83.2 (3)
C2—Fe1—C4—C3	−38.0 (2)	C22—Fe2—C25—C24	−167.9 (3)
C2—Fe1—C4—C5	81.4 (2)	C22—Fe2—C25—C26	−48.3 (4)
C3—Fe1—C4—C5	119.4 (3)	C24—Fe2—C25—C26	119.5 (3)
C5—Fe1—C4—C3	−119.4 (3)	C26—Fe2—C25—C24	−119.5 (3)
C6—Fe1—C4—C3	56.2 (4)	C27—Fe2—C25—C24	−82.0 (3)
C6—Fe1—C4—C5	175.6 (3)	C27—Fe2—C25—C26	37.6 (3)
C7—Fe1—C4—C3	87.2 (3)	C28—Fe2—C25—C24	−38.3 (3)
C7—Fe1—C4—C5	−153.4 (2)	C28—Fe2—C25—C26	81.3 (3)
C8—Fe1—C4—C3	129.7 (2)	C20—Fe2—C26—C25	74.2 (3)
C8—Fe1—C4—C5	−110.9 (2)	C20—Fe2—C26—C27	−166.7 (3)
C9—Fe1—C4—C3	169.0 (2)	C21—Fe2—C26—C25	114.8 (3)
C9—Fe1—C4—C5	−71.6 (3)	C21—Fe2—C26—C27	−126.1 (3)
C1—Fe1—C5—C4	−119.1 (3)	C22—Fe2—C26—C25	157.2 (2)
C2—Fe1—C5—C1	37.6 (2)	C22—Fe2—C26—C27	−83.6 (3)
C2—Fe1—C5—C4	−81.5 (2)	C23—Fe2—C26—C25	−168.2 (3)
C3—Fe1—C5—C1	81.7 (2)	C23—Fe2—C26—C27	−49.1 (5)
C3—Fe1—C5—C4	−37.4 (2)	C24—Fe2—C26—C25	−37.1 (2)
C4—Fe1—C5—C1	119.1 (3)	C24—Fe2—C26—C27	82.0 (3)
C7—Fe1—C5—C1	170.6 (3)	C25—Fe2—C26—C27	119.1 (4)
C7—Fe1—C5—C4	51.5 (4)	C27—Fe2—C26—C25	−119.1 (4)
C8—Fe1—C5—C1	−155.6 (2)	C28—Fe2—C26—C25	−81.1 (3)
C8—Fe1—C5—C4	85.3 (2)	C28—Fe2—C26—C27	38.0 (3)
C9—Fe1—C5—C1	−113.1 (2)	C19—Fe2—C27—C26	−168.9 (3)
C9—Fe1—C5—C4	127.8 (2)	C19—Fe2—C27—C28	−50.5 (5)
C10—Fe1—C5—C1	−73.6 (3)	C21—Fe2—C27—C26	73.6 (3)
C10—Fe1—C5—C4	167.3 (2)	C21—Fe2—C27—C28	−168.0 (3)
C1—Fe1—C6—C7	169.5 (2)	C22—Fe2—C27—C26	114.1 (3)
C1—Fe1—C6—C10	−70.2 (3)	C22—Fe2—C27—C28	−127.5 (3)
C2—Fe1—C6—C7	127.9 (2)	C23—Fe2—C27—C26	156.9 (3)
C2—Fe1—C6—C10	−111.7 (2)	C23—Fe2—C27—C28	−84.7 (3)
C3—Fe1—C6—C7	83.5 (3)	C24—Fe2—C27—C26	−80.8 (3)
C3—Fe1—C6—C10	−156.1 (2)	C24—Fe2—C27—C28	37.6 (3)
C4—Fe1—C6—C7	45.5 (4)	C25—Fe2—C27—C26	−37.6 (3)
C4—Fe1—C6—C10	165.8 (3)	C25—Fe2—C27—C28	80.8 (3)
C7—Fe1—C6—C10	120.4 (3)	C26—Fe2—C27—C28	118.4 (4)
C8—Fe1—C6—C7	−38.5 (2)	C28—Fe2—C27—C26	−118.4 (4)
C8—Fe1—C6—C10	82.0 (2)	C19—Fe2—C28—C24	−84.2 (3)
C9—Fe1—C6—C7	−82.2 (2)	C19—Fe2—C28—C27	156.4 (3)
C9—Fe1—C6—C10	38.2 (2)	C20—Fe2—C28—C24	−49.4 (6)
C10—Fe1—C6—C7	−120.4 (3)	C20—Fe2—C28—C27	−168.8 (4)
C2—Fe1—C7—C6	−74.5 (3)	C22—Fe2—C28—C24	−168.4 (2)
C2—Fe1—C7—C8	167.7 (2)	C22—Fe2—C28—C27	72.2 (4)
C3—Fe1—C7—C6	−114.2 (2)	C23—Fe2—C28—C24	−127.0 (3)
C3—Fe1—C7—C8	128.0 (2)	C23—Fe2—C28—C27	113.6 (3)
C4—Fe1—C7—C6	−157.1 (2)	C24—Fe2—C28—C27	−119.4 (5)
C4—Fe1—C7—C8	85.1 (3)	C25—Fe2—C28—C24	37.7 (3)
C5—Fe1—C7—C6	168.2 (3)	C25—Fe2—C28—C27	−81.7 (3)
C5—Fe1—C7—C8	50.3 (4)	C26—Fe2—C28—C24	81.5 (3)
C6—Fe1—C7—C8	−117.9 (3)	C26—Fe2—C28—C27	−38.0 (3)
C8—Fe1—C7—C6	117.9 (3)	C27—Fe2—C28—C24	119.4 (5)
C9—Fe1—C7—C6	80.8 (2)	C11—N1—C12—C13	−81.2 (4)
C9—Fe1—C7—C8	−37.1 (2)	C11—N1—C12—C17	156.6 (3)
C10—Fe1—C7—C6	36.9 (2)	C12—N1—C11—C1	178.4 (3)
C10—Fe1—C7—C8	−81.0 (2)	C18—N2—C17—C12	160.1 (3)
C1—Fe1—C8—C7	173.5 (3)	C18—N2—C17—C16	−74.6 (4)
C1—Fe1—C8—C9	53.4 (4)	C17—N2—C18—C19	168.3 (3)
C3—Fe1—C8—C7	−71.8 (3)	C11—C1—C2—Fe1	−122.8 (3)
C3—Fe1—C8—C9	168.1 (2)	C11—C1—C2—C3	177.7 (3)
C4—Fe1—C8—C7	−111.0 (2)	C5—C1—C2—C3	−0.5 (4)
C4—Fe1—C8—C9	128.9 (2)	C11—C1—C5—C4	−178.3 (3)
C5—Fe1—C8—C7	−153.9 (2)	Fe1—C1—C11—N1	49.8 (4)
C5—Fe1—C8—C9	86.0 (3)	C2—C1—C11—N1	141.3 (3)
C6—Fe1—C8—C7	38.4 (2)	C5—C1—C11—N1	−40.8 (5)
C6—Fe1—C8—C9	−81.8 (3)	Fe1—C1—C5—C4	59.1 (2)
C7—Fe1—C8—C9	−120.1 (3)	Fe1—C1—C2—C3	−59.4 (3)
C9—Fe1—C8—C7	120.1 (3)	C5—C1—C2—Fe1	58.9 (2)
C10—Fe1—C8—C7	82.0 (2)	C2—C1—C5—C4	0.0 (4)
C10—Fe1—C8—C9	−38.1 (2)	C11—C1—C5—Fe1	122.6 (3)
C1—Fe1—C9—C8	−153.0 (2)	C2—C1—C5—Fe1	−59.1 (2)
C1—Fe1—C9—C10	88.3 (2)	C1—C2—C3—Fe1	59.5 (2)
C2—Fe1—C9—C8	174.2 (3)	C1—C2—C3—C4	0.9 (4)
C2—Fe1—C9—C10	55.5 (4)	Fe1—C2—C3—C4	−58.6 (3)
C4—Fe1—C9—C8	−69.8 (3)	C2—C3—C4—C5	−0.9 (4)
C4—Fe1—C9—C10	171.5 (2)	C2—C3—C4—Fe1	59.0 (3)
C5—Fe1—C9—C8	−110.3 (2)	Fe1—C3—C4—C5	−59.9 (3)
C5—Fe1—C9—C10	131.0 (2)	Fe1—C4—C5—C1	−59.7 (2)
C6—Fe1—C9—C8	81.0 (2)	C3—C4—C5—C1	0.6 (4)
C6—Fe1—C9—C10	−37.8 (2)	C3—C4—C5—Fe1	60.3 (3)
C7—Fe1—C9—C8	37.2 (2)	Fe1—C6—C10—C9	−59.4 (3)
C7—Fe1—C9—C10	−81.5 (2)	C7—C6—C10—Fe1	58.3 (2)
C8—Fe1—C9—C10	−118.7 (3)	C7—C6—C10—C9	−1.1 (4)
C10—Fe1—C9—C8	118.7 (3)	C10—C6—C7—C8	1.4 (4)
C1—Fe1—C10—C6	131.2 (2)	Fe1—C6—C7—C8	60.1 (3)
C1—Fe1—C10—C9	−110.2 (2)	C10—C6—C7—Fe1	−58.7 (2)
C2—Fe1—C10—C6	87.8 (2)	Fe1—C7—C8—C9	59.5 (3)
C2—Fe1—C10—C9	−153.6 (2)	C6—C7—C8—C9	−1.2 (4)
C3—Fe1—C10—C6	50.2 (4)	C6—C7—C8—Fe1	−60.7 (2)
C3—Fe1—C10—C9	168.8 (3)	C7—C8—C9—Fe1	−59.3 (3)
C5—Fe1—C10—C6	172.8 (2)	C7—C8—C9—C10	0.5 (4)
C5—Fe1—C10—C9	−68.6 (3)	Fe1—C8—C9—C10	59.8 (3)
C6—Fe1—C10—C9	118.6 (3)	C8—C9—C10—Fe1	−59.4 (3)
C7—Fe1—C10—C6	−37.0 (2)	Fe1—C9—C10—C6	59.8 (2)
C7—Fe1—C10—C9	81.6 (2)	C8—C9—C10—C6	0.4 (4)
C8—Fe1—C10—C6	−80.9 (2)	N1—C12—C13—C14	−174.5 (3)
C8—Fe1—C10—C9	37.8 (2)	C17—C12—C13—C14	−53.7 (5)
C9—Fe1—C10—C6	−118.6 (3)	N1—C12—C17—N2	−59.7 (4)
C20—Fe2—C19—C18	−121.8 (4)	N1—C12—C17—C16	173.0 (3)
C20—Fe2—C19—C23	118.2 (3)	C13—C12—C17—N2	179.5 (3)
C21—Fe2—C19—C18	−159.0 (4)	C13—C12—C17—C16	52.3 (5)
C21—Fe2—C19—C20	−37.2 (2)	C12—C13—C14—C15	56.5 (5)
C21—Fe2—C19—C23	81.0 (2)	C13—C14—C15—C16	−57.8 (6)
C22—Fe2—C19—C18	157.6 (3)	C14—C15—C16—C17	57.7 (5)
C22—Fe2—C19—C20	−80.6 (2)	C15—C16—C17—N2	−179.5 (3)
C22—Fe2—C19—C23	37.6 (2)	C15—C16—C17—C12	−55.1 (5)
C23—Fe2—C19—C18	120.0 (4)	N2—C18—C19—Fe2	179.3 (2)
C23—Fe2—C19—C20	−118.2 (3)	N2—C18—C19—C20	87.8 (4)
C24—Fe2—C19—C18	−7.2 (3)	N2—C18—C19—C23	−90.3 (4)
C24—Fe2—C19—C20	114.6 (2)	Fe2—C19—C20—C21	59.1 (3)
C24—Fe2—C19—C23	−127.2 (2)	C18—C19—C20—Fe2	122.0 (4)
C25—Fe2—C19—C18	−47.5 (4)	C18—C19—C20—C21	−179.0 (3)
C25—Fe2—C19—C20	74.3 (3)	C23—C19—C20—Fe2	−59.6 (2)
C25—Fe2—C19—C23	−167.5 (2)	C23—C19—C20—C21	−0.6 (4)
C27—Fe2—C19—C18	70.8 (5)	Fe2—C19—C23—C22	−59.2 (3)
C27—Fe2—C19—C20	−167.4 (3)	C18—C19—C23—Fe2	−122.0 (4)
C27—Fe2—C19—C23	−49.2 (4)	C18—C19—C23—C22	178.8 (3)
C28—Fe2—C19—C18	35.8 (4)	C20—C19—C23—Fe2	59.6 (2)
C28—Fe2—C19—C20	157.6 (2)	C20—C19—C23—C22	0.4 (4)
C28—Fe2—C19—C23	−84.2 (3)	Fe2—C20—C21—C22	59.7 (3)
C19—Fe2—C20—C21	−119.8 (3)	C19—C20—C21—Fe2	−59.2 (2)
C21—Fe2—C20—C19	119.8 (3)	C19—C20—C21—C22	0.5 (4)
C22—Fe2—C20—C19	82.1 (2)	Fe2—C21—C22—C23	59.7 (3)
C22—Fe2—C20—C21	−37.7 (2)	C20—C21—C22—Fe2	−59.9 (3)
C23—Fe2—C20—C19	38.0 (2)	C20—C21—C22—C23	−0.3 (4)
C23—Fe2—C20—C21	−81.7 (3)	Fe2—C22—C23—C19	59.7 (3)
C24—Fe2—C20—C19	−84.1 (2)	C21—C22—C23—Fe2	−59.8 (3)
C24—Fe2—C20—C21	156.2 (2)	C21—C22—C23—C19	−0.1 (4)
C25—Fe2—C20—C19	−126.5 (2)	Fe2—C24—C25—C26	−59.3 (3)
C25—Fe2—C20—C21	113.7 (2)	C28—C24—C25—Fe2	60.4 (3)
C26—Fe2—C20—C19	−167.6 (2)	C28—C24—C25—C26	1.1 (5)
C26—Fe2—C20—C21	72.6 (3)	Fe2—C24—C28—C27	59.6 (3)
C28—Fe2—C20—C19	−49.4 (5)	C25—C24—C28—Fe2	−60.8 (3)
C28—Fe2—C20—C21	−169.1 (4)	C25—C24—C28—C27	−1.2 (5)
C19—Fe2—C21—C20	37.5 (2)	Fe2—C25—C26—C27	−59.5 (3)
C19—Fe2—C21—C22	−81.3 (2)	C24—C25—C26—Fe2	59.0 (3)
C20—Fe2—C21—C22	−118.8 (3)	C24—C25—C26—C27	−0.5 (5)
C22—Fe2—C21—C20	118.8 (3)	Fe2—C26—C27—C28	−59.9 (3)
C23—Fe2—C21—C20	81.1 (2)	C25—C26—C27—Fe2	59.7 (3)
C23—Fe2—C21—C22	−37.7 (2)	C25—C26—C27—C28	−0.2 (5)
C24—Fe2—C21—C20	−51.3 (5)	Fe2—C27—C28—C24	−59.2 (3)
C24—Fe2—C21—C22	−170.1 (3)	C26—C27—C28—Fe2	60.1 (3)
C25—Fe2—C21—C20	−84.8 (3)	C26—C27—C28—C24	0.9 (5)
C25—Fe2—C21—C22	156.4 (2)		

### Hydrogen-bond geometry (Å, °)

**Table:**

*D*—H···*A*	*D*—H	H···*A*	*D*···*A*	*D*—H···*A*
N1—H1A···N2	0.90	2.36	2.848 (4)	114
